# Specific Roles of MicroRNAs in Their Interactions with Environmental Factors

**DOI:** 10.1155/2012/978384

**Published:** 2012-10-31

**Authors:** Juan Wang, Qinghua Cui

**Affiliations:** ^1^Department of Biomedical Informatics, Peking University Health Science Center, Beijing 100191, China; ^2^MOE Key Lab of Cardiovascular Sciences, Peking University, Beijing 100191, China; ^3^Institute of Systems Biomedicine, Peking University, Beijing 100191, China

## Abstract

MicroRNAs (miRNAs) have emerged as critical regulators of gene expression by modulating numerous target mRNAs expression at posttranscriptional level. Extensive studies have shown that miRNAs are critical in various important biological processes, including cell growth, proliferation, differentiation, development, and apoptosis. In terms of their importance, miRNA dysfunction has been associated with a broad range of diseases. Increased number of studies have shown that miRNAs can functionally interact with a wide spectrum of environmental factors (EFs) including drugs, industrial materials, virus and bacterial pathogens, cigarette smoking, alcohol, nutrition, sleep, exercise, stress, and radiation. More importantly, the interactions between miRNAs and EFs have been shown to play critical roles in determining abnormal phenotypes and diseases. In this paper, we propose an outline of the current knowledge about specific roles of miRNAs in their interactions with various EFs and analyze the literatures detailing miRNAs-EFs interactions in the context of various of diseases.

## 1. Introduction

MicroRNAs (miRNAs) are regulatory RNAs that are 20–30 nucleotides long that bind the 3′-untranslated regions of target mRNAs [1–3]. miRNAs have emerged as critical regulators of gene expression by modulating the expression of numerous target mRNAs mainly at the posttranscriptional level [4]. Since partial or imperfect complementarity of an miRNA to a target mRNA can lead to translational repression, a single miRNA has the ability of regulating a large number of genes [5]. miRNAs have been shown to play a role in regulating a wide range of biological processes, such as cell growth, proliferation, differentiation, development, and apoptosis [6]. In terms of their importance, dysfunction of miRNAs has been associated with various diseases [7–9]. In contrast to the wealth of publications about their biological effects, the information about specific regulations of miRNAs has comparatively lagged behind.

**Figure 1:**
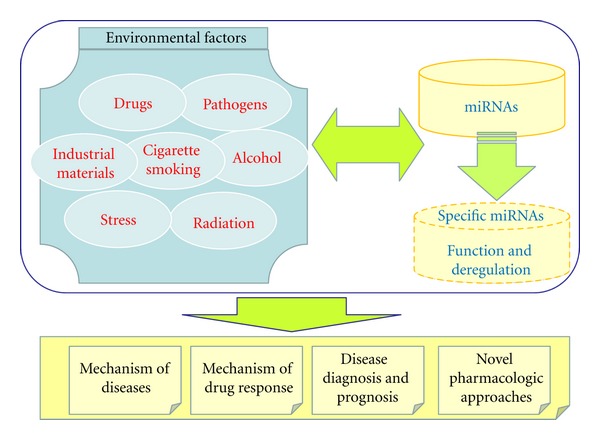
Significant roles of miRNAs-EFs interactions.

The phenotype of an organism is determined by the complex interactions between genetic factors (GFs) and environmental factors (EFs). EFs have been shown to contribute tremendously to the formation and development of many diseases, especially complex diseases [10–12]. The interactions between GFs and EFs, often hypothesized to be mediated by epigenetic mechanisms, modulate the reproductive fitness of an organism, its response to external stimuli and health [13]. Similar to other GFs, miRNAs have complex interactions with a wide spectrum of EFs [14]. Recently, increased number of studies have shown that miRNAs can functionally interact with a variety of EFs including drugs [15, 16], industrial materials [17, 18], virus and bacterial pathogens [19, 20], alcohol [21–23], cigarette smoking [24–26], nutrition [27, 28], sleep [29], exercise [30, 31], stress [32, 33], and radiation [34]. The interactions between miRNAs and these EFs play critical roles in determining phenotypes. The illustration of the specific roles of miRNAs and the patterns of miRNA deregulation in the miRNAs-EFs interactions can help to discover the mechanisms of diseases and drug response therefore providing insights into disease diagnosis, prognosis and novel pharmacologic approaches (Figure 1). In this paper, we propose an outline of the current understanding about specific roles of miRNAs when they are interacting with various EFs and analyze the literatures studying the miRNAs-EFs interactions in the context of diseases.

## 2. Drugs

Previous studies had highlighted the post-transcriptional modifications mediated by miRNAs in drug treatment, drug sensitivity, and drug addiction.

Several groups have shown the altered miRNAs profiles in anticancer drugs treatment, including all-trans-retinoic acid [35, 36], hydroxycamptothecin [37, 38], imatinib [16, 39–41], 5-fluorouracil [42, 43], gemcitabine [44–47], cisplatin [48–52], fludarabine [53], lithium [54–56], epigallocatechin gallate [57], and curcumin [58–60]. Garzon et al. studied the effects of all-trans-retinoic acid (ATRA) treatment on the expression of miRNAs in acute promyelocytic leukemia patients as well as in cell lines by using a miRNA microarray platform and quantitative real-time polymerase chain reaction (qRT-PCR). Their results showed upregulation of miR-15a, 15b, 16-1, 223, 342 107 and let-7a-3, let-7c, let-7d and downregulation of miR-181b in ATRA treatment. Among the upregulated miRNAs, miR-107 was predicted to target NFI-A, a gene involved in the regulatory loop with the participation of miR-223 and C/EBPa during granulocytic differentiation [35].

Drug insensitivity or resistance is a major obstacle for successful cancer therapy. Increasing evidence suggested that miRNAs modulate cellular sensitivity to anticancer reagents. Wu et al. showed significantly different sensitivities of the six gastric cancer cell lines to hydroxycamptothecin (HCPT). The levels of 25 miRNAs were shown to be deregulated in the HCPT-resistant gastric cancer cells, including miR-200 family, miR-196a, 338, 126, 31, 98, 7 and let-7 g. The target genes of these miRNAs are involved in cancer development, progression, and chemosensitivity [37]. Ali et al. evaluated the effects of curcumin or its analogue difluorinated-curcumin (CDF) alone or in combination with gemcitabine on cell viability and apoptosis in gemcitabine-sensitive as well as gemcitabine-resistant pancreatic cancer (PC) cell lines [58]. They showed downregulation of miR-200 and upregulation of miR-21 (an indicator of tumor aggressiveness) in gemcitabine-resistant cells in comparison to gemcitabine-sensitive cells. In addition, gemcitabine sensitivity can be induced in PC cells through the modulation of miR-200 and miR-21 expression by curcumin or its analogue CDF. With the application of anti-miR-21 oligonucleotide, Li et al. were able to use anti-miR-21 oligonucleotide to enhance the chemosensitivity of leukemic HL60 cells to arabinosylcytosine [61]. In the study performed by Ji et al. in 2009, the expression patterns of miRNAs in patients with hepatocellular carcinoma (HCC), their survival, and response to interferon alfa were examined [62]. They found a higher expression of miR-26a and miR-26b in non-tumor liver tissue in female than in male HCC patients. They also showed significantly decreased levels of miR-26 expression in comparison to the paired noncancerous tissues. Furthermore, cancer patients with tumors of low miR-26 level had shorter overall survival but better response to interferon therapy than those with tumors of high levels of miR-26. These results suggested the association between the miR-26 expression level and the survival of HCC patients as well as their response to adjuvant interferon alfa therapy [62].

Drug abuse induces persistent structural and functional changes in the mesolimbic dopaminergic system and leads to the progression of patients toward the high-risk drug-seeking behavior and relapse. Much emerging evidence suggests that drug-induced neuroplasticity is regulated by gene expression and post-transcriptional regulation [63, 64]. Chandrasekar and Dreyer had shown that a chronic usage of cocaine decreased miR-124 and let-7d levels and increased the expression of miR-181a in mesolimbic pathway [65]. They also proposed a complex regulatory pathway involving miRNAs in cocaine-mediated neuronal adaptations [66]. Evidence has suggested that cross-regulation between transcription factors and miR-212/132 played a role in drug addiction [67]. MiR-132 and miR-212 are tandem miRNAs whose expression is necessary for the proper development, maturation, and function of neurons. Deregulation of miR-212/132 had been associated with several neurological disorders [67]. It has been shown that overexpression of miR-212 induces CREB activity, the negative modulator of the reward response to cocaine, therefore exerts protective function against cocaine addiction by reducing the responsiveness to the motivational properties of the drug [68]. The X-linked transcriptional repressor methyl CpG binding protein 2 (MeCP2), known for its role in the neurodevelopmental disorder Rett syndrome, is emerging as an important regulator of neuroplasticity in postmitotic neurons. MeCP2 regulates cocaine intake through homeostatic interactions with miR-212 to control the effects of cocaine on striatal brain-derived neurotrophic factor (BDNF) levels. As MeCP2 levels are correlated to BDNF expression, which controls cocaine intake, miR-212 could fine-tune the responses of patients to drug abuse by both increasing CREB signaling and decreasing BDNF expression, leading to decreased cocaine intake [69].

## 3. Industrial Materials

Many industrial materials have toxicity and may alter developmental pathways and cell processes through epigenetic mechanisms. Bisphenol A (BPA) is an industrial plasticizer widely used as coating for food cans and plastic water bottles. The frequency of BPA exposure has steadily increased during recent years [18]. Whiting and colleagues investigated the effects of BPA exposure on miRNAs in human placental cell lines using miRNA microarray and qRT-PCR analysis. MiR-146a was shown to be the only miRNA validated by qRT-PCR as being significantly upregulated in both 3A and HTR-8 cell lines with BPA treatment [17]. It was also demonstrated that stable overexpression of miR-146a in 3A placental cell line leads to a significant decrease in cell proliferation, a result consistent with several other studies [70, 71]. In addition, over-expression of miR-146a in placental cell lines led to increased sensitivity of these cells to bleomycin, the DNA damaging agent, suggesting that the targets of miR-146a may be involved in the DNA damage response.

Jardim et al. investigated the pollutant-mediated regulations of miRNAs in airway epithelial cells with diesel exhaust particles (DEP), the largest source of emitted airborne particulate matter (PM).They showed that DEP exposure significantly modulated the miRNA expression profile in human airway epithelial cell lines. Specifically, 197 out of 313 (62.9%) detectable miRNAs had been either upregulated or downregulated by 1.5 fold. Molecular network analysis of putative targets of the 12 mostly altered miRNAs suggested that DEP exposure was associated with inflammatory responsive pathways and a strong tumorigenic disease signature. These results clearly demonstrated that alteration of miRNA expression profiles by environmental pollutants such as DEP could modify cellular processes by regulation of gene expression and subsequently lead to disease pathogenesis [72]. In a similar study, Bollati et al. discovered the significant increases of the expression of miR-222 and miR-21 in peripheral blood leukocytes from foundry workers with exposure to metal-rich particulate matter [73].

## 4. Virus and Other Pathogens

**Figure 2:**
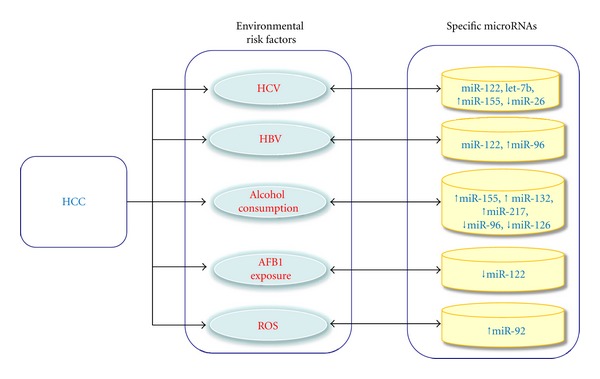
Specific miRNAs are associated with different subgroups of HCC associated with various environmental risk factors. ↑ indicates up-regulation; ↓ indicates down-regulation; HCC: hepatocellular carcinoma; HCV: hepatitis C virus; HBV: hepatitis B virus; AFB1: aflatoxin B1; ROS: reactive oxygen species.

miRNAs have been known to play a critical role in the life cycle of retroviruses and a few oncogenic viruses, including HIV [74, 75], reticuloendotheliosis virus strain T (REV-T) [76], influenza A virus [77], Epstein-Barr virus [78, 79], human T-cell leukemia virus type 1 (HTLV-1) [80, 81], gammaretrovirus XMRV [82], cytomegalovirus (HCMV) [83, 84], Hepatitis B virus (HBV) [85, 86], Hepatitis C virus (HCV) [87, 88], and human papilloma virus (HPV) [89]. These viruses regulate viral replication in the host cells with the mediation of specific miRNAs. HCC is the fifth most common cancer worldwide. The greatest risk factor for HCC is the existence of cirrhosis, most commonly induced by HBV or HCV infections as well as factors such as alcohol consumption, oxidative stress, and aflatoxin or arsenic exposure. Specific miRNAs have been shown to be involved in subgroups of HCC caused by different environmental risk factors. Examinations of miRNAs functions and their expression are important for HCC diagnosis, treatment, and prognosis (Figure 2). Several host cell factors including liver-specific miR-122 have been shown to be involved in virus translation, replication, and production. Jopling et al. observed that sequestration of miR-122 in liver cells could result in marked loss of autonomously replicating hepatitis C viral RNAs. MiR-122 has been shown to increase HCV abundance in HCV replication models by interacting with the 5′ noncoding region (NCR) of the HCV genome [90]. MiR-122 has also been shown to stimulate HCV translation by enhancing the association of ribosome with viral RNA at an early initiation stage [91]. Studies by Lanford and colleagues suggested that miR-122 was essential for the accumulation of HCV RNA in chronically HCV-infected chimpanzees by inhibiting miR-122 through a specific locked nucleic acid (LNA) oligonucleotide [87]. Qiu et al. also suggested a role of miR-122 in the regulation of HBV replication by downregulating the Heme oxygenase-1 (HO-1) [92]. These results suggest that miR-122 may serve as a target for antiviral intervention.

Human T-lymphotropic virus type 1 (HTLV-1) is the etiologic agent to which the prolonged exposure can cause the adult T-cell leukemia of CD4+ T-cell origin, a severe and fatal lymphoproliferative disease. Pichler et al. reported the deregulation of several miRNAs, including miR-21, 24, 146a, 155, and 223 in HTLV-1-transformed cells. The expression pattern of these miRNAs presented a uniform phenotype in HTLV-transformed cells in comparison to HTLV-negative control cells. Studies suggested miR-146a could be directly attributed to oncoprotein Tax via NF-*κ*B-mediated transactivation in HTLV signaling pathways [81].

Recent studies characterized modifications of host miRNAs expression following infection by exclusively extracellular (*Helicobacter pylori*) or intracellular (*Salmonella enterica*) gram-negative bacteria, gram-positive bacteria (*Listeria monocytogenes*) and other pathogens such as *Mycobacterium*, *Francisella* species, and *Plasmodium berghei* [20, 93, 94].

## 5. Cigarette Smoking

Throughout one's life course, a number of exposures can affect an individual's development, health, and overall quality of life. One of the most common, potentially hazardous environmental exposures that negatively influence health and development is cigarette smoke exposure [24]. Maccani et al. investigated the effects of maternal cigarette smoking during pregnancy on differential expression of miRNAs in the placenta. The data suggested that maternal cigarette smoking during pregnancy was associated with the down-regulation of miR-16, 21, and 146a. They further identified two components, nicotine and benzo [a]pyrene as the specific miRNA modulators in cigarette smoking [26, 95].

Xi et al. investigated the effects of cigarette smoke condensate (CSC) on miRNA expression and function in both normal human respiratory epithelial cells and lung cancer cells [96]. Their results showed that the exposure to CSC induced the expression of miR-31 in both normal respiratory epithelial cells and lung cancer cells. Further studies showed that the overexpression of miR-31 led to increased proliferation and tumorigenicity in lung cancer cells, whereas knockdown of miR-31 expression significantly decreased lung cancer cell proliferation [96]. These results suggested that miR-31 was responsive to cigarette smoke exposure in both normal respiratory epithelia and lung cancer cells and might play a role as an oncomir in lung cancer carcinogenesis pathway [96]. Schembri et al. investigated whole-genome miRNA expression in bronchial airway epithelium from regular smokers and nonsmokers. They observed that 28 miRNAs were differentially expressed, the majority of which were downregulated in the airway epithelium of regular smokers including miR-218 [97]. Several studies have also been performed to characterize the effects of environmental cigarette smoke (ECS) (also called “passive,” “secondhand,” or “sidestream” cigarette smoke) on miRNA expression. Izzotti et al. found that the most greatly downregulated miRNAs belonged to miRNA families which had been previously shown to regulate a number of key biological processes, including stress response, proliferation, angiogenesis, and apoptosis [98]. Furthermore, they showed that exposure to ECS upregulated 2.9% of genes and 9.7% of proteins in the same tissue, suggesting that the ECS exposure-induced down-regulation of miRNAs may lead to increased protein levels of genes which are negatively regulated by these miRNAs [98].

## 6. Alcohol

Neurotoxicity-mediated pathophysiological conditions could manifest diseases or disabilities such as Parkinson's and Alzheimer's which have debilitating implications. Neurotoxicity as well as brain injury can be caused by external agents including drugs andalcohol, and miRNAs have been shown to play a pivotal role in these processes [99]. Maternal ethanol consumption during pregnancy can lead to a stereotypic cluster of fetal craniofacial, cardiovascular, skeletal, and neurological defects that are collectively termed the fetal alcohol spectrum disorder (FASD) [22]. The developing brain is an important and vulnerable target for ethanol. In 2007, Sathyan et al. obtained the first evidence showing that miRNAs mediated the teratogenic effects by ethanol treatment [100]. The accumulated data support the following conclusions. (1) Ethanol appears to affect a relatively small subset of expressed miRNAs. (2) Ethanol appears to affect miRNAs which are normal components of the cellular repertoire for a given stage of differentiation. As an example, miR-124 is sufficient to direct fetal neural stem cells (NSCs) toward a neuronal lineage (22,101). (3) Ethanol appears to affect increasing numbers of miRNAs along the developmental stage. (4) MiRNAs targeted by ethanol exhibit developmental stage-specific sensitivity. (5) Some ethanol-sensitive miRNAs exert their functions over multiple developmental stages, for example, miR-9 exhibits ethanol sensitivity at multiple stages of development, expanding from the embryo through fetus to the adulthood [22, 101, 102]. In order to illustrate the mechanisms of ethanol regulation of miRNAs, Sathyan et al. reported that GABAA receptors and nicotinic acetylcholine receptors could mediate certain ethanol effects on miRNAs expression, respectively [100, 101]. It has been shown that several ethanol-sensitive miRNAs are localized on chromosomal regions that are susceptible to epigenetic modifications. Ligand-gated ion channel receptors and epigenetic modifications have been shown to be potential mediators of the effects of ethanol on fetal miRNAs [22]. Ethanol and nicotine are often coabused. Balaraman et al. studied their combined effects on fetal neural development, particularly on NSCs. Their results showed that ethanol suppressed the expression of known ethanol-sensitive miRNAs (miR-9, 21, 153, and 335) and miR-140-3p, while nicotine at the concentrations attained by cigarette smokers could induce a dose-related induction in these miRNAs. These data suggest that concurrent exposure to ethanol and nicotine disrupts miRNA regulatory networks that are important for NSC maturation [101].

Alcohol-dependence can lead to long-term changes of gene expression in brain. Tapocik et al. examined the potential roles of miRNAs in persistent gene expression regulation of the medial prefrontal cortex (mPFC) of the rats with a history ofalcohol-dependence.Their results showed that more than 41 rat miRNAs and 165 mRNAs in the mPFC were significantly altered after chronicalcoholexposure. Categories of gene ontology differential expression indicated that these miRNAs served in functional processes commonly associated with neurotransmission, neuroadaptation, and synaptic plasticity. Their results demonstrated a significant shift inmiRNAexpression pattern in the mPFC following a history of alcohol-dependence. MiRNAs may play a pivotal role in the reorganization of synaptic connections and long-term neuroadaptations in alcohol-dependence due to their functions of global regulation of multiple target transcripts [23].

Chronicalcoholabuse can cause liver damage, including inflammation, fatty liver, fibrosis, cirrhosis, and HCC. Bala and Szabo showed thatalcoholcould induce miR-155 and miR-132 expression in liver as well as in isolated hepatocytes and Kupffer cells ofalcohol-fed mice [103].Yin et al. showed that miR-217 could promote ethanol-induced fat accumulation in hepatocytes by downregulating SIRT1 [104]. Ladeiro et al. suggested the miRNA deregulations may be associated with alcohol consumption and HBV infection in HCC. Their studies indicated the correlation between miR-126 down-regulation and alcohol abuse-related HCC in comparison to the other HCCs (Figure 2). Their miRNAs profiling is the first global genomic approach that enables us to differentiate the alcohol abuse-related HCC from the other HCC tumors [21].

## 7. Stress

Cells frequently encounter conditions that can lead to stress. A few well-characterized stressors include hyperthermia, hypoxia, ATP depletion, and oxidative stress [105, 106]. In order to survive and adapt to stressful conditions, all mammalian cells have evolved a molecular defense reaction termed the cellular stress response (CSR). Recent studies have suggested that miRNAs participate in the CSR, such as hypothermia [107], hyperthermia [108], hypoxia [109–112], folate deficiency, and arsenic exposure [33].

Wilmink et al. studied the specific group of deregulated miRNAs in the cells exposed to hyperthermia. Their results showed that the heat could induce upregulation of miRNAs including miR-125b, 382, and 452 and downregulation of miRNAs including miR-138, 7, and 196b. Several thermally-regulated miRNAs (miR-452, 382, and 378) expresses only in cells exposed to hyperthermia [108].

Hypoxia is an essential feature of the neoplastic microenvironment. Tumors with extensive low oxygen level tend to exhibit poor prognosis and resistance to conventional therapy [109]. Ivan's group identified a set of hypoxia-regulated miRNAs (HRMs), including miR-21, 23a, 23b, 24, 26a, 26b, 27a, 30b, 93, 103, 103, 106a, 107, 125b, 181a, 181b, 181c, 192, 195, 210, and 213 [112]. In addition, a set of miRNAs were down-regulated in hypoxic cells, including miR-15b, 16, 19a, 20a, 20b, 29b, 30b, 30e-5p, 101, 141, 122a, 186, 320, and 197 [113, 114]. Saito et al. suggested the involvement of the miR-130 family in hypoxia-induced expression of hypoxia inducible factor 1*α* (HIF-1*α*). MiR-130 family has been identified to target DDX6 mRNA, which is a component of the P-bodies, and facilitate the translation of HIF-1*α* during hypoxia [110].

Dietary folate deficiency has been linked to developmental anomalies as well as increased risk for a number of cancers [115]. Low dietary folate levels lead to decreased DNA stability through misincorporation of uracil and subsequent DNA damage during the repair process as well as to increased genomic hypomethylation [116]. Arsenic exposure, similar to ionizing radiation and folate deficiency, has been linked to a variety of human cancers [117]. Marsit et al. examined the in vitro effect of folate deficiency and arsenic exposure on the expression pattern of 385 known human miRNAs using microarray analysis and they discovered alterations of specific miRNA profiles. The miRNAs that were significantly altered by 6-day folate deficiency treatment included miR-181b, 182, 222, 345, 181a, 205, 145, 99a, 125b, 130b, 221, 22, 191, 103, 107, 34a, 198, 183, 146, 422b, 210, 24, and 361 [33]. These results suggested that aberrant phenotypes might result from alterations in key miRNAs expression at critical stages in development and tumorigenesis due to altered folate status. Reduced dietary folate could lead to decreased level of S-adenosyl methionine, which is the required methyl-group donor for all cellular methylation reactions, including those for DNA and proteins such as histones [33]. The altered miRNAs expression profiles may be related to the altered methyl-donor pool and the subsequent epigenetic alterations at the DNA or histone code level, resulting in changes of miRNA and gene expression [33]. The miRNAs that were significantly altered by 6-day arsenic exposure included miR-210, 22, 34a, 221, and 222 [33], all of which were also altered by folate deficiency. Similar effects on genomic methylation status have also been reported for arsenic [118]. These results supported the hypothesis that arsenic may alter one-carbon metabolism and therefore cause downstream epigenetic effects.

## 8. Radiation

DNA damage can be caused by multiple stressors including ionizing radiation, UV exposure, reactive oxygen species, and many DNA damaging chemicals, such as doxorubicin and camptothecin [119–121]. MiRNA-mediated gene regulation has been contributed to the mechanisms of DNA-damage caused by UV exposure and ionizing radiation. Studies by Pothof et al. suggested that the cellular responses to UV-induced DNA damage were also regulated at the post-transcriptional level by miRNAs. UV damage has been shown to cause cell-cycle-dependent relocalization of Ago2 into stress granules and changes of miRNA expression. Both events of miRNA-expression alteration and stress-granule formation occurred within the first hour following genotoxic stress, which suggested miRNAs-mediated gene regulation happened earlier than most transcriptional responses. The functionality of the miRNAs response was illustrated by the UV-inducible miR-16 which down-regulated checkpoint-gene CDC25a and regulated cell proliferation [122].

Ionizing radiation (IR) has been widely used in cancer therapy and biological studies. It disrupts cellular homeostasis through multiple mechanisms including the alteration of gene expression profile [123]. Shin et al. investigated the effects of IR on miRNA expression profile in the human lung carcinoma cell line A549. Microarray analysis identified 12 and 18 miRNAs in 20- and 40-Gy-exposed A549 cells, respectively, with more than 2-fold changes in their expression levels. Target prediction for IR-responsive miRNAs suggested that these miRNAs might target genes which are related to apoptosis, regulation of cell cycle, and DNA damage and repair. The results suggested that the expression levels of miRNAs could be affected by radiation and they might be involved in the regulation of radiation responses [123]. Niemoeller et al. investigated IR-induced miRNA expression profiles in six malignant cell lines. Their results showed that IR could cause 2-3 fold change of the expression level of miRNAs known to be involved in the regulation of cellular processes such as apoptosis, proliferation, invasion, local immune response, and radioresistance (e.g., miR-1285, 24-1, 151-5p, and let-7i). Moreover, they discovered several new miRNAs that are radiation-responsive (e.g., miR-144) [34].

Exposure to high-dose radiation causes ionization in the molecules of living cells, leads to DNA damage, and promotes tumor formation. On the other hand, low-dose radiation induces the beneficial effects on organisms, named as hormesis. Cha et al. identified responses of miRNAs to low- or high-dose *γ*-irradiation in the human lymphoblast line IM9. They discovered that IR exposure could induce changes in specific miRNA expression in a dose-dependent manner and provided evidence that low-dose radiation could suppress the progression of malignant cancer by controlling miRNA expression [124].

## 9. Conclusion and Future Directions

Environmental exposures including drugs, industrial materials, cigarette smoking, alcohol, stress, and radiation may cause a number of deleterious effects on health, development, and survival. Increased research has discovered the roles of miRNAs in the responses to environmental exposures under various conditions. The interactions of miRNAs and EFs have been associated with abnormal phenotypes and diseases. For example, specific miRNAs have been identified for different subgroups of HCC with various environmental risk factors. Investigation into the specific roles of miRNAs in the interactions between miRNAs and EFs may help uncovering the mechanisms of disease. These studies may potentially lead to the development of useful indicators of toxic exposure or novel biomarkers for diseases diagnosis. Studies of miRNAs in the disease context may also contribute to the development of novel pharmacologic approaches. The recent development of miRNA derivatives with increased stability and binding efficiency, including anti-microRNA oligonucleotides (AMOs) and LNAs served such purpose [87, 109]. As an example, targeting an HRM which plays a survival role in hypoxia may provide a new angle in targeting a notoriously refractory fraction of tumor cells [109]. Anti-miR-21 oligonucleotide has been shown to enhance the chemosensitivity of leukemic HL60 cells to arabinosylcytosine by inducing apoptosis [61]. Treatment of chronically infected chimpanzees with a LNA-modified oligonucleotide (SPC3649) complementary to miR-122 led to long-lasting suppression of HCV viremia [87]. Furthermore, manipulations of selected miRNAs could synergize with conventional therapies in the treatment of various diseases. Finally, insilico analysis and modeling may help studying patterns among miRNAs, EFs, and diseases and identifying novel strategies for disease diagnosis, treatment, and prognosis [125].
